# The spatial epidemiology of leprosy in Kenya: A retrospective study

**DOI:** 10.1371/journal.pntd.0007329

**Published:** 2019-04-22

**Authors:** Fatihiyya Wangara, Hillary Kipruto, Oscar Ngesa, James Kayima, Enos Masini, Joseph Sitienei, Faith Ngari

**Affiliations:** 1 Department of Health Services, Kwale County Government, Kwale, Kenya; 2 Health systems and services, World Health Organization, Nairobi, Kenya; 3 Department of Mathematics and Informatics, Taita Taveta University, Voi, Kenya; 4 Department of Medicine, Makerere University, Kampala, Uganda; 5 Communicable Disease Control, World Health Organization, Nairobi, Kenya; 6 National Strategic Program, Ministry of Health, Nairobi, Kenya; 7 National Tuberculosis Leprosy and Lung Disease Program, Ministry of Health, Nairobi, Kenya; EPFL, SWITZERLAND

## Abstract

**Background:**

Leprosy elimination defined as a registered prevalence rate of less than 1 case per 10,000 persons was achieved in Kenya at the national level in 1989. However, there are still pockets of leprosy in some counties where late diagnosis and consequent physical disability persist. The epidemiology of leprosy in Kenya for the period 2012 through to 2015 was defined using spatial methods.

**Methods:**

This was a retrospective ecological correlational study that utilized leprosy case based data extracted from the National Leprosy Control Program database. Geographic information system and demographic data were obtained from Kenya National Bureau of Statistics (KNBS). Chi square tests were carried out to check for association between sociodemographic factors and disease indicators. Two Spatial Poisson Conditional Autoregressive (CAR) models were fitted in WinBUGS 1.4 software. The first model included all leprosy cases (new, retreatment, transfers from another health facility) and the second one included only new leprosy cases. These models were used to estimate leprosy relative risks per county as compared to the whole country i.e. the risk of presenting with leprosy given the geographical location.

**Principal findings:**

Children aged less than 15 years accounted for 7.5% of all leprosy cases indicating active leprosy transmission in Kenya. The risk of leprosy notification increased by about 5% for every 1 year increase in age, whereas a 1% increase in the proportion of MB cases increased the chances of new leprosy case notification by 4%. When compared to the whole country, counties with the highest risk of leprosy include Kwale (relative risk of 15), Kilifi (RR;8.9) and Homabay (RR;4.1), whereas Turkana had the lowest relative risk of 0.005.

**Conclusion:**

Leprosy incidence exhibits geographical variation and there is need to institute tailored local control measures in these areas to reduce the burden of disability.

## Author summary

Leprosy is a chronic bacterial disease that mainly affects the nerves. If untreated, it may cause progressive and permanent damage to the skin, nerves, limbs, and eyes leading to physical disability. Through use of a combination of drugs, Kenya was able to declare the disease as eliminated in the year 1989. However, there are still pockets of leprosy in some Kenyan counties where physical disability persists, mainly due to late diagnosis. To be able to curb this disease, control measures must be intensified, especially in the counties reporting more cases. We used data of the leprosy cases reported in Kenya for the period 2012 through to 2015 in order to describe geographical variation and factors influencing this variation. More than half of the registered cases had visible physical disability. The risk of leprosy notification increased with an increase in age as well as the severity of disease. We estimated that people living in Kwale, Kilifi and Homabay counties are 15, 9, and 5 times respectively more at risk of leprosy as compared to the whole country. Given the limited resources, it’s therefore paramount that high risk counties be initially targeted for control, with a focus on early diagnosis.

## Introduction

In 1991, the World Health Assembly passed a resolution to “eliminate” leprosy as a public health problem by the year 2000. Elimination, defined as a registered prevalence rate of less than 1 case per 10 000 persons, was realized globally in the year 2000 and in most countries by 2005 [1]. This achievement was driven by the utilization of multiple drug therapy (MDT) as a strategy for elimination of leprosy. More than 16 million leprosy patients have been treated globally over the past 20 years and the prevalence rate of the disease has dropped by 99%: from 21.1 per 10 000 in1983 to 0.2 per 10 000 persons in 2015 [2]. All countries with a population of one million or more have achieved the elimination of leprosy as a public health problem at the national level [1]. Despite the definite gains in control of leprosy, on-going transmission continues to be documented. More than 200,000 new leprosy cases are detected and reported annually from 121 countries [3]. This number has been fairly stable in the past 8 years with India, Brazil and Indonesia accounting for 81% of all new cases.

Kenya is in the post elimination phase of leprosy control, having achieved the WHO elimination target of less than 1 case per 10,000 people in 1989. The number of new reported leprosy cases in the country declined steadily from 6,558 in 1986 to 131 cases in 2015 [4]. Despite the low number of reported cases, leprosy continues to cause high morbidity among those infected with 48% of new cases notified in 2013 having advanced disease with disability grade 1 and 2. In 2014, 133 Leprosy cases were notified, majority (90%) being multibacilary (MB) patients. This advanced form of the disease implies localized infection which continues to be spread in the communities as individuals stay for longer periods before being diagnosed [5]. Additionally, childhood cases accounted for 11% and 2% of the cases diagnosed in the year 2014 and 2015 respectively, indicating ongoing active transmission [4,5].

Geographical variations are a striking feature of leprosy at every level. In Kenya, for example, most new leprosy cases have been documented in Kwale, Kilifi, Kisumu, Siaya, Homabay and Busia counties [5], located in the south east and western parts of the country. These geographical patterns may indicate important risk factors that remain to be elucidated and whose recognition could be useful in control of the disease. In addition, the recognition of tailor made leprosy control activities e.g. active case finding which improves the cost–effectiveness of control programs, considering that a reduced disease burden in terms of the number of new cases is likely to define the nature of leprosy in the future. We set out to describe the geospatial distribution of leprosy cases and to determine factors influencing leprosy notification in the 47 counties in Kenya as a way of providing data to plan for leprosy elimination strategies.

## Methods

### Study design and setting

This is a retrospective cross sectional study that covered all the 47 administrative units (counties) in Kenya. Kenya covers an approximate area of 591,971 km^2^ with an estimated population of 43 million people in 2014 [6]. The county is the most important unit of administration in provision of social services in Kenya’s decentralized system of governance. Leprosy care and control in Kenya is fully integrated in the national primary health care network and involves most government health facilities, faith based organization, communities and private health care units. Health care workers in these facilities are responsible for case finding, infection control and treatment of leprosy patients. At the county and sub-county levels Tuberculosis (TB) and leprosy coordinators are responsible for providing technical assistance and supervision to the health facilities. This cadre of healthcare workers is also responsible for aggregating data at the sub county level and updating the web-based surveillance system (TIBU) making leprosy case-based data available at the national level. The National Tuberculosis, Leprosy and Lung Disease Program (NTLD-P) designs standard data collection and reporting tools for all the levels of reporting (national, county, sub-county and facility) [7].

### Data sources

The study utilized secondary data from the TIBU system i.e. retrospective extraction of leprosy case-based notification data as well as geospatial data from Kenya National Bureau of Statistics (KNBS). The key patient variables collected in the TIBU system and of relevance to this study include, sex (male/female), age in years, classification of patient (MB/PB) and disability grade at diagnosis (0, 1, 2). This disability grading is done according to the WHO disability grading scale [8] outlined in Table 1.

**Table 1:** WHO disability grading scale.

Body Area	Signs / Symptoms	Disability Grade
**Hands and Feet**	No anesthesia, no visible deformity or damage	0
	Anesthesia present, but no visible deformity or damage	1
	Visible deformity or damage present	2
**Eyes**	No eye problem due to leprosy, no evidence of visual loss	0
	Eye problems due to leprosy present but vision not severely affected (can count fingers at 6 meters)	1
	Severe visual impairment (inability to count fingers at 6 meters), lagophthalmos, irridocyclitis and corneal opacities	2

The case-based data was downloaded as an excel file. Considering the relatively few numbers of leprosy cases notified in the country, and the geographic distribution, counties with the highest number of notified cases were purposively sampled for data verification. These included Kwale, Killifi, Malindi, Kisumu, Siaya, Homabay, Busia and Bungoma counties. Two health facilities from among those which reported any case of leprosy within the study period were randomly selected from each county and data in the facility register (considered to be the primary data source) matched to data available in the online TIBU system. We included all notified cases of leprosy within a 4-year period (2012 to 2015). Observations missing any one or more requisite variables were excluded.

### Statistical methods

The variables of interest for description of the trend were sex, the number of leprosy cases reported over the four year period, annual new case detection rate (per 100,000 population), disease classification of patient (MB/PB), disability grade at diagnosis (0, 1, 2) and age in years. Microsoft excel was used to summarize data in graphs and frequency tables to illustrate changes in leprosy case detection over the years. Univariate analysis (chi square tests) to explore the relationship between types of leprosy notified and sociodemographic/ disease factors were carried out in STATA version 11.2.

To describe the geospatial distribution of notified leprosy cases across the 47 counties, a Bayesian approach was used [9,10]. Two separate spatial Poisson Conditional Autoregressive (CAR) models were fitted i.e. one for all cases (new, retreatment, transfers from other health facilities) and another including only new leprosy cases. The covariates included in both models were population density, proportion of < 15 year olds among newly diagnosed cases, proportion of newly diagnosed cases with Grade 2 Disability (G2D), proportion of MB cases among new cases, median age of leprosy cases (years) and sex ratio per county. The relationship between notified leprosy cases and the covariates were characterized by spatial random effects. Spatially unstructured random effects were assumed to be normally distributed whereas spatially structured random effects were assigned a conditional autoregressive prior and the corresponding precision parameters given non-informative gamma distributed priors. Two counties were said to be neighbors if they shared a border. Bayesian inference was used to estimate the parameters in the model with Markov Chain Monte Carlo (MCMC) technique. The models were implemented using WinBUGS version 14 and MCMC convergence of all model parameters assessed by checking trace plots. The relative risks per county were then mapped.

### Ethics

Ethical approval to carry out the study was obtained from Kenyatta National Hospital/University of Nairobi Ethics and Research Committee. The data extracted from the National Leprosy Control Program database was anonymized and no reference made to the patient names, serial numbers or address/ immediate neighborhoods or any other person identifiable variable. All data were password protected hence only authorized persons had access to it.

## Results

**Fig 1:**
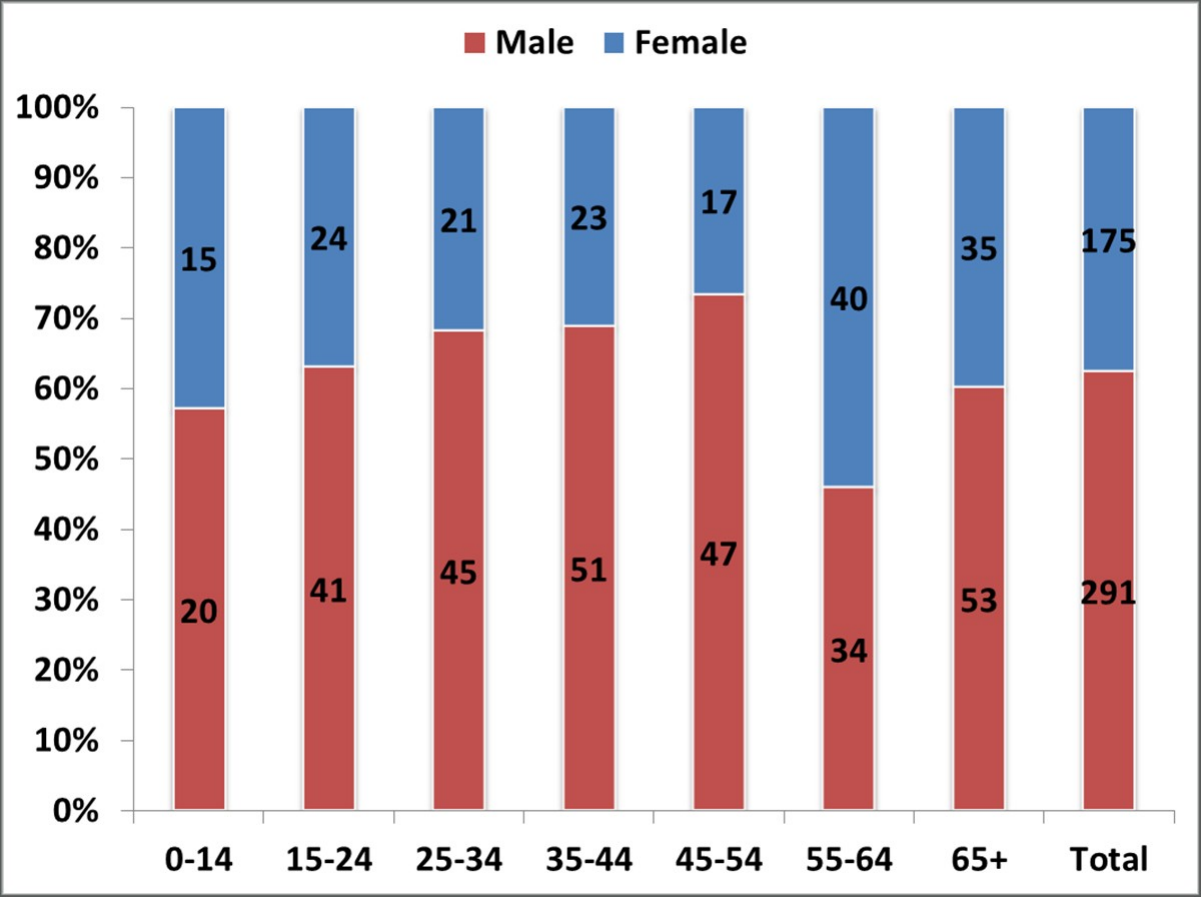
Age distribution of notified leprosy cases.

The data is the TIBU system matched that of the sampled healthcare facilities. A total of 467 leprosy cases were notified in Kenya over the four year period distributed among 28 counties. Out of these, 291 (62%) were males and 176 (38%) females. There were 380 new cases representing about 81% of all notified cases. Fig 1 shows the age-distribution of notified leprosy cases. Out of the notified cases, 88 of them were 65 years and older, accounting for about 19% of the total cases. There were 35 childhood cases (0–14 years) accounting for 7.5% of the total cases. Generally, the number of cases reported increased with increasing age.

**Fig 2:**
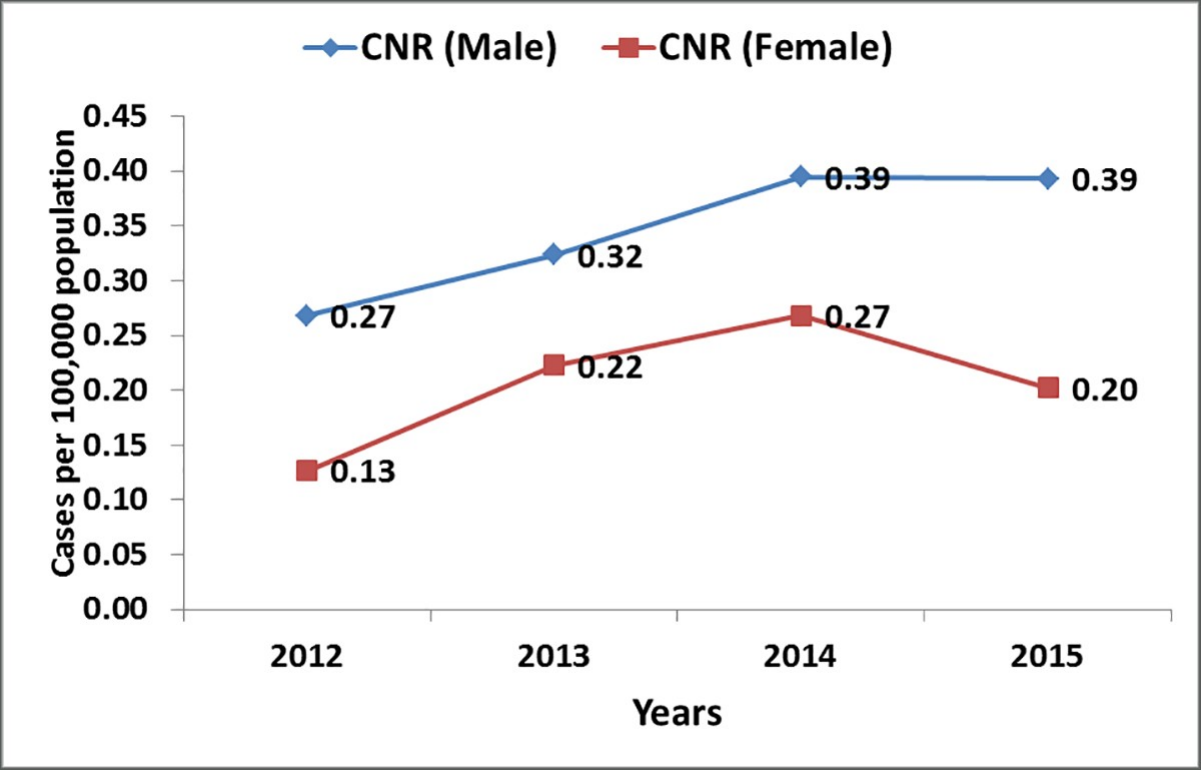
Annual leprosy case notification rate (CNR) by sex.

Fig 2 shows the annual case notification rates for leprosy. Throughout the reporting period, case notification rate was persistently higher among males than females. The highest rates were documented in 2014 with 0.39 and 0.27 cases per 100,000 population among males and females respectively; while the lowest rates were noted in 2012 with 0.27 and 0.13 cases per 100,000 population among males and females respectively.

**Fig 3:**
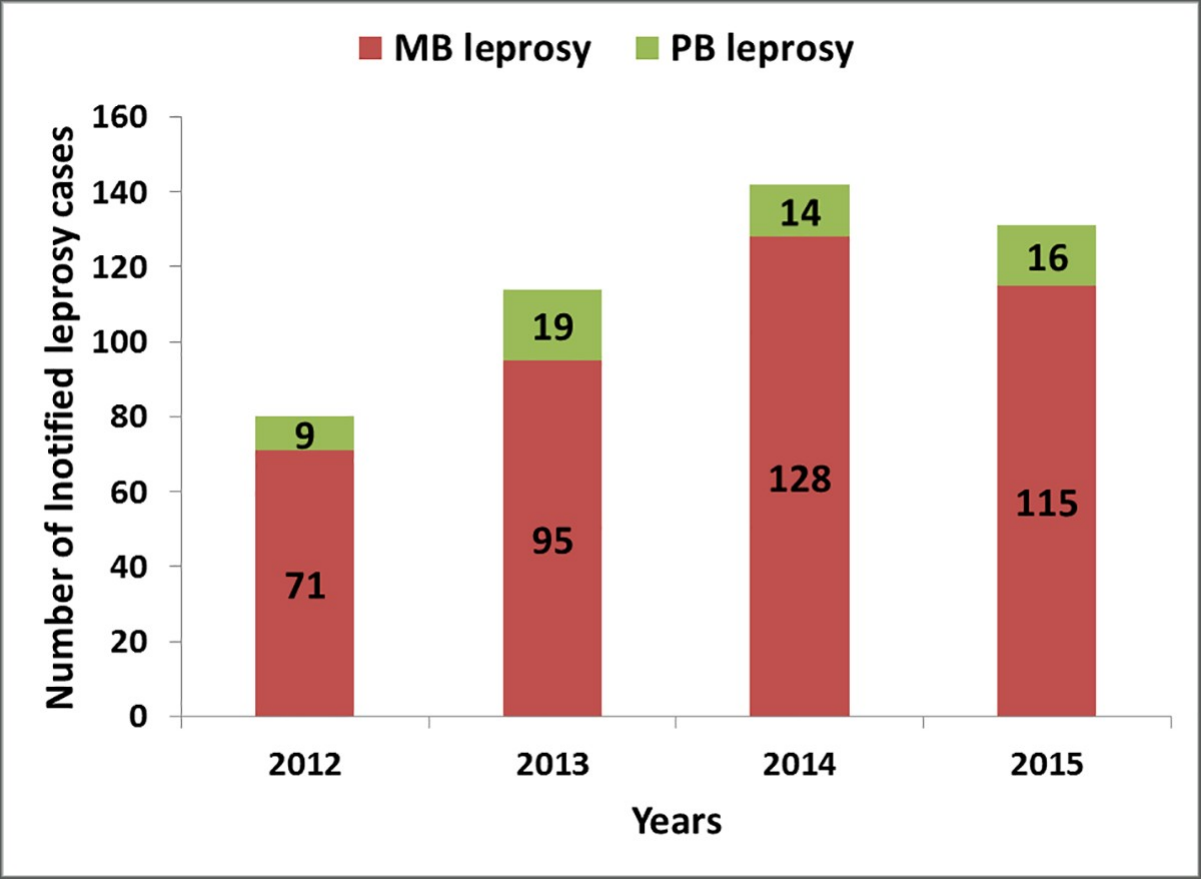
Annual leprosy cases notified by disease severity.

Fig 3 shows the proportion of MB and paucibacillary (PB) leprosy among notified cases. Of the total number of cases, 409 (88%) were of the MB type. This pattern of reporting was relatively similar throughout the reporting years.

Disability was present in more than half (52.9%) of cases for whom disability grading had been undertaken. Grade 1 disability accounted for 34.5% while Grade 2 accounted for 18.4% of all cases. The association between leprosy type and some demographic and disease indicators is shown in Table 2. Chi square test showed that patient age was significantly associated with disease severity.

**Table 2:** Association between leprosy type and sociodemographic/ disease factors.

Variables	Type of Leprosy		p-value
	MB cases n (%)	PB cases n (%)	
**Year (of diagnosis)** 2012 2013 2014 2015	71 (89) 95 (83) 128 (90) 115 (88)	9 (11) 19 (17) 14 (10) 16 (12)	0.415
**Sex** Female Male	150 (85) 259 (89)	26 (15) 32 (11)	0.231
**Age (Years)** 0–14 15–64 65+	25 (71) 304 (89) 80 (90)	10 (29) 39 (11) 8 (9)	0.008
**Type of Patient** New Not new	329 (87) 80 (92)	51 (13) 7 (8)	0.170
**Disability Grade** Grade 0 Grade 1 Grade 2 Not Done	166 (83) 135 (92) 69 (88) 39 (89)	33 (17) 11 (8) 9 (12) 5 (11.36)	0.091

MB: Multibacillary; PB: Paucibacillary

### Spatial analysis

When all cases were considered, age proved to be the only significant predictor, with the risk of leprosy notification increasing by about 5% for every 1 year increase in age. Among newly diagnosed cases, the proportion of MB cases was a significant predictor for leprosy notification. A one percent increase in the proportion of MB cases increased the leprosy risk by approximately 4%. Table 3 summarizes the model results.

**Table 3:** Spatial poisson regression results.

Covariate	Adjusted OR (95% CI)	
	New cases	All cases
Sex ratio	3.58E^-7^ (2.2E^-15^, 4.78)	2.0E-4 (5.8E^-11^, 1.645)
Population density	1.00 (1.00, 1.00)	1.00 (0.009, 1.00)
Proportion < 15 years	1.02 (0.97, 1.07)	1.02 (0.97, 1.07)
Proportion of Grade 2 Disability	1.004 (0.98, 1.03)	1.003 (0.98, 1.03)
Proportion of MB leprosy.	1.04 (1.003, 1.096)	1.02 (0.99, 1.06)
Median age	1.028 (0.96, 1.096)	1.05 (1.0007, 1.11)

MP: Multibacillary

#### Geographical mapping

**Fig 4:**
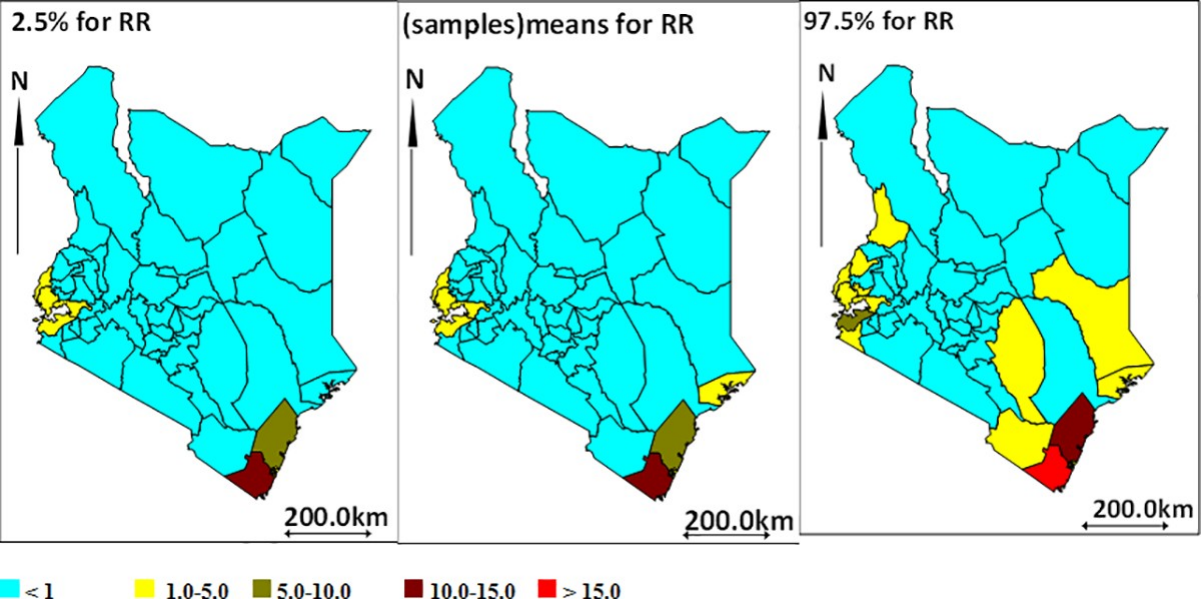
Leprosy relative risks (all cases) and 95% CI; lower bound, mean and upper bound respectively.

**Fig 5:**
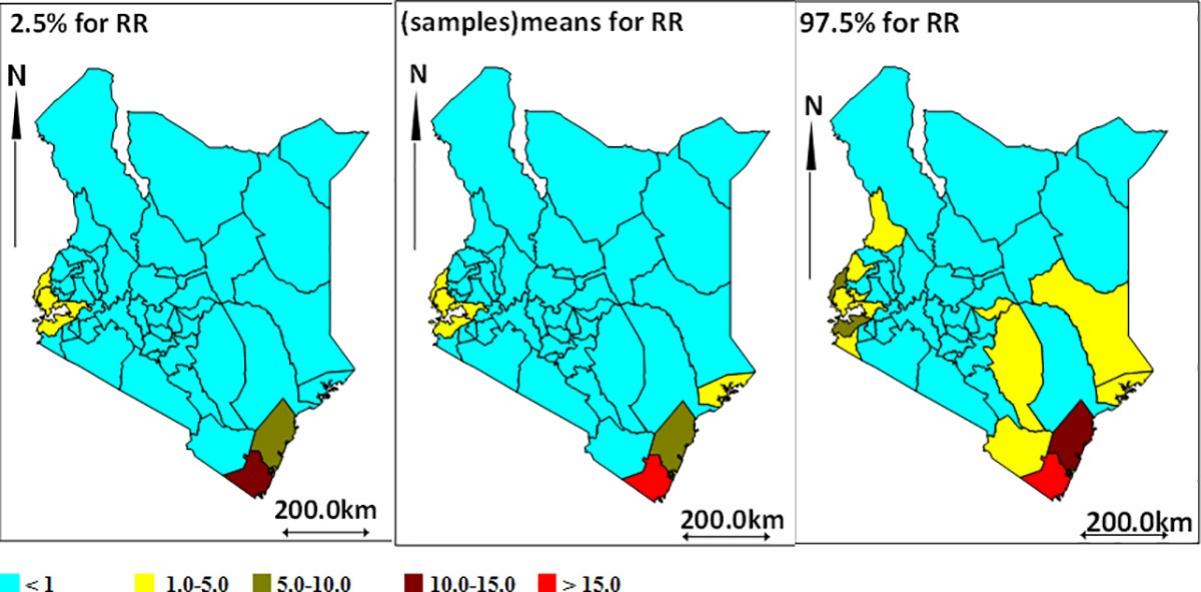
Leprosy relative risks (new cases) and 95% CI; lower bound, mean and upper bound respectively.

Fig 4 shows the relative risks of leprosy per county when all cases are considered, whereas Fig 5 illustrates relative risk for new leprosy cases. The western (on the shores of Lake Victoria) and South Eastern (coastal) regions of the country have higher leprosy risks as compared to the rest of the country. Compared to the rest of the country, the counties with the highest relative risk of leprosy include Kwale (RR;15), Kilifi (RR;8.9) and Homabay (RR;4.1), whereas Turkana had the lowest relative risk of 0.005. Similarly, these regions had the highest risk of new cases with Kwale (RR;16) Kilifi (RR;8.6) and Homabay (RR;3.7) compared to the rest of the county. Turkana remained the county with the lowest risk of new case (RR;0.003).

## Discussion

Our results suggest the higher and lower risk areas of leprosy occurrence in mainland Kenya. Areas proximal to Lake Victoria in the west (Homa Bay and Siaya Counties); and coastal area (Kwale and Kilifi) are the hotspots for leprosy occurrence and transmission. Leprosy transmission remains active in Kenya as evidenced by the substantial number of cases aged below 15 years. Childhood cases are usually associated with recent active foci of transmission, given leprosy’s long periods of incubation i.e. 2–5 years for PB disease and 5–10 years or sometimes longer for MB disease [11]. The large proportion of notified cases with MB disease is also concerning as it is the major risk factor for leprosy transmission [12].

We documented an increase in cases notified with age. This is a similar trend in both low and high density transmission areas where a peak in cases is highly correlated with the life expectancy [13,14]. While most of the incidental infection occurs in young adulthood, the long incubation period of disease and the fact that disability rather than mortality occurs in cases, combine to contribute to the high numbers in late adulthood [15].

The high proportion of cases with disability indicates late diagnosis of leprosy. The high numbers of disability have a negative socioeconomic impact on communities that harbor the patients not only because of the symptoms but also the stigma attached to the condition [16]. Community engagement in ensuring early diagnosis, instituting multidrug therapy and tackling stigma has been suggested as a way of reducing the psychosocial and economic impact of leprosy [17]. It is possible that adopting similar strategies would have an impact in reducing disability in highly endemic counties in Kenya.

The annual case notification rates increased from 2012 and peaked in 2014 followed by a drop in 2015. Leprosy control activities have not been a priority in Kenya. In the year 2014, funds were availed to conduct an active case finding in some counties like Kwale and Kisumu generating considerably high numbers of cases. This kind of financial support is not consistent and the health system in Kenya largely relies on passive surveillance of leprosy. The low index of suspicion among health workers is detrimental to the process. The results also indicate that more males than female cases were reported across the ages. From the results, it remains unclear whether there is a significantly higher risk in males or if it is merely due to a biased case ascertainment. A similar scenario has been documented in most countries where leprosy still exists [18]. However, in most African countries, while the men make up the bigger proportion of leprosy cases, the outcomes- disability and death- seem to be poorer in women [19,20]. The nature of our study does not allow us to reach conclusions on gender differences related to access to health which may play a role in determining outcomes. The Global Leprosy Strategy (2016–2020) advocates for special focus on women and children. The leprosy control program should therefore ensure equity in access to health services.

The regression models revealed that significant risk factors for leprosy incidence in Kenya include the age (for all cases) and the proportion of MB disease among newly diagnosed cases. These findings are consistent with existing literature; given MB contact is a high risk factor hence the higher the proportion, the higher the probability of contact. In addition, with increasing age, so does the probability of manifestation of an earlier infection.

As depicted by the spatial maps, the coastal areas and those near to Lake Victoria have high relative risks. In other countries proximity- and especially bathing in open water bodies has been shown to catalyze transmission [21]. In addition, counties with high relative risks tend to be close to each other. This suggests geographical variation in either the risk factors, population based factors and/or health system factors.

One of the fundamental strategies towards improving the surveillance system proposed in the Kenya National Strategic Plan for Leprosy (2015–2018) was to map leprosy cases to identify the hot spots. This work contributes substantially to this process and therefore guides appropriate and cost-effective programmatic interventions. These would focus the constrained resources on high risk counties herein defined as Kwale, Kilifi, Homa Bay, Siaya, Busia, Mombasa, Kisumu and Lamu. In these counties, the following approaches should be implemented: i) sensitization of health care workers and community health volunteers on leprosy, specifically early symptoms of the disease ii) tracing and screening household contacts of children (below 15 years of age) and those with newly diagnosed multibacillary leprosy and iii) conducting active case finding ensuring no biased case ascertainment; women, children and other vulnerable populations to be included. The respective county health departments should be proactive and allocate resources for leprosy control. A well planned surveillance system will not only improve treatment outcomes, but also strengthen monitoring and evaluation by generating data that is comparable over time.

This study is not without limitation. Firstly, leprosy case notification rate is used as a proxy for disease incidence and does not capture the true incidence of disease as not all leprosy cases may be registered at a health facility. However, the data generated, even if a proxy, is instrumental in planning interventions. Secondly, secondary data sources, whose collection we had no control over, usually has problems with completeness. To address this problem, we compared primary data at randomly selected health centers and we found it to be complete. Future studies could focus on the effects of various socio‐economic and environmental risk factors for the high occurrence of the disease in the clustered areas and analyses of case isolates will enable anti‐microbial and strain‐specific factors to be considered. We conclude that there is evidence of geographical variation in occurrence of leprosy cases with clustering in western and coastal areas of Kenya. There is also evidence of active leprosy transmission and significant disability among the cases. This work will guide tailored policies to address leprosy control.

## Supporting information

S1 Checklist STROBE checklist. (DOC)

S1 Detailed Methodology Relative risk estimation and mapping. (DOCX)
