# Supplementary material for: The spatial epidemiology of leprosy in Kenya: A retrospective study
Source: PLoS Negl Trop Dis. 2019 Apr 22;13(4):e0007329. doi: 10.1371/journal.pntd.0007329 (PMC6497316; doi:10.1371/journal.pntd.0007329)
Supplement: S1 Checklist — (DOC) [file pntd.0007329.s001.doc]

**STROBE Statement— The spatial epidemiology of leprosy in Kenya: A retrospective study**

|  |  |
| --- | --- |
| Title and abstract | Abstract, paragraph 1-4 |
| Background/rationale | Introduction, paragraph 2 |
| Objectives | Introduction, paragraph 3 |
| Study design | Methods, paragraph 1 |
| Setting | Methods, paragraph 1 |
| Participants | Methods, paragraph 3 |
| Variables | Methods, paragraph 2  Methods, paragraph 5 |
| Data sources/ measurement | Methods, paragraph 2 |
| Bias | Discussion, paragraph 8 |
| Study size | Methods, paragraph 3 |
| Quantitative variables | Methods, paragraph 5 |
| Statistical methods | Methods, paragraph 4-5 |
| Participants | Methods, paragraph 2 |
| Descriptive data | Results, paragraph 1 |
| Outcome data | Results, paragraph 2-4 |
| Main results | Results, paragraph 5-6 |
| Other analyses | Results, paragraph 5-6 |
| Key results | Results, paragraph 6 |
| Limitations | Discussion, paragraph 8 |
| Interpretation | Discussion, paragraph 1-7 |
| Generalisability | Discussion, paragraph 8 |
