# Supplementary material for: The spatial epidemiology of leprosy in Kenya: A retrospective study
Source: PLoS Negl Trop Dis. 2019 Apr 22;13(4):e0007329. doi: 10.1371/journal.pntd.0007329 (PMC6497316; doi:10.1371/journal.pntd.0007329)
Supplement: S1 Detailed Methodology — (DOCX) [file pntd.0007329.s002.docx]

**The spatial epidemiology of leprosy in Kenya: A retrospective study**

**Bayesian Spatial Model**

A generalized linear mixed model assuming a Poisson distribution of the outcome variable (leprosy incidence) was used i.e.

$y_{i}\sim poisson\left( \mu_{i} \right)$

Where *y_i_* is the number of leprosy cases reported in region (county) *i*.

The relationship between leprosy cases notified and the covariates were characterized by spatial random effects. The GLMM is of the form:

$$Log\left( \mu_{i} \right)=Log \left( E_{i} \right)+\beta_{0}+\sum_{j=1}^{k} \beta_{j}X_{ij}+u_{i}+v_{i}$$

Where;

*u_i_,*and *v_i_* represent spatially unstructured and spatially structured random effects respectively.

X_ij_ represents the j^th^ covariate for county i.

*β_j_* represents the parameter vector of the covariates *X_ij_*, and *β_0_* is the model intercept.

*E_i_* is the expected number of leprosy cases in county *i.* In this model *i* ranges from 1 to 47. The *E_i_* was calculated with indirect standardization of age and sex as follows:

$$Age specific E_{i}= \left[ \frac{y_{sl}(m)}{P_{sl}(m)}\times p_{il}(m) \right]+\left[ \frac{y_{sl}(f)}{P_{sl}(f)}\times p_{il}(f) \right]$$

*y_sl_(m)* = number of male leprosy cases in age group *l* in the standard population (the whole country population was used as the standard population).

*y_sl_(f)* = number of female leprosy cases in age group *l* in the standard population

*P_sl_(m)* = total number of males in age group *l* in the standard population

*P_sl_(f)* = total number of females in age group *l* in the standard population

*p_il_(m)* = total number of males in age group *l* in county *i*

*p_il_(f)* = total number of females in age group *l* in county *i*

Therefore;

$$E_{i}=\sum(\left[ \frac{y_{sl}(m)}{P_{sl}(m)}\times p_{il}(m) \right]+\left[ \frac{y_{sl}(f)}{P_{sl}(f)}\times p_{il}(f) \right])$$

The covariates found to be significant were used to calculate county specific relative risks (RR) for mapping;

$$RR_{i}=exp(\beta_{0}+\sum_{j=1}^{k} \beta_{j}X_{ij}+u_{i}+v_{i})$$

**Parameter estimation:**

Bayesian inference was used to estimate the parameters in the model with Markov Chain Monte Carlo (MCMC) technique. Non-informative uniform and normally distributed priors were assigned for the model intercept and covariate parameter vector respectively i.e. $\beta_{0} \sim U \left( -\infty, +\infty\right)$

$\beta_{j} \sim Normal{(0,\delta}_{\beta}^{2})$

Spatially unstructured random effects were assumed to be normally distributed i.e. $u_{i} \sim Normal\left( 0, \delta_{u}^{2} \right)$ whereas spatially structured random effects were assigned a conditional autoregressive prior i.e.$v_{i} \sim CAR(\delta_{v}^{2})$, and the corresponding precision parameters given non-informative gamma distributed priors. Two counties were said to be neighbors if they shared a boarder implying that the conditional distribution of each *v_i_* given the rest, is;

$v_{i}\sim N\left( {\sum u_{i}\in N_{i}, \atop d_{i}}, \delta^{2} \right)$ Where *_di_* is the number of neighbors of county *i*, and *N_i_* is the set of neighbors of county *i.*

The models were implemented using WinBUGS version 14 and MCMC convergence of all models parameters assessed by checking trace plots. The Deviance Information Criterion (DIC) was used to select best fitting model (smallest DIC).

**Variables**

The variables for the spatial model and disease risk mapping:

- Number of leprosy cases per county
- Population density
- Proportion of < 15 year olds among newly diagnosed cases
- Proportion of newly diagnosed cases with G2D
- Proportion of MB cases among new cases
- Median age of leprosy cases (Years)
- County sex ratio

**WinBUGS code for fitting the spatial Poisson CAR full model**

model

{

# Likelihood

for(i in 1:N)

{

Y[i]~dpois(mu[i])

log(mu[i])<-log(Eall[i])+beta0+beta1*sexratio[i]+beta2*density[i]+beta3*propunder15[i]+

beta4*propG2D[i]+beta5*propMB[i]+beta6*medianage[i]+

u[i]+v[i]

RR[i]<-exp(beta0+beta1*sexratio[i]+beta2*density[i]+beta3*propunder15[i]+beta4*propG2D[i]+beta5*propMB[i]+beta6*medianage[i]+u[i]+v[i]) # County specific relative risk

# Prior on unstructured random effects

u[i]~dnorm(0,precu)

}

# CAR prior for spatial random effects

v[1:N]~car.normal(adj[],weights[],num[],precv)

for(k in 1:sumNumNeigh)

{

weights[k]<-1

}

# Other priors

beta0~dflat()

beta1~dnorm(0,0.00001)

beta2~dnorm(0,0.00001)

beta3~dnorm(0,0.00001)

beta4~dnorm(0.0,00001)

beta5~dnorm(0,0.00001)

beta6~dnorm(0,0.00001)

precu~dgamma(0.01,0.01) # priors on precision

precv~dgamma(0.5,0.0005)

sigmav<-sqrt(1/precv) # Standard deviation of v

sigmau<-sqrt(1/precu) # Standard deviation of u

}

# Initial values

list(precu=1,precv=1,beta0=0,beta1=0,beta2=0,beta3=0,beta4=0,beta5=0,beta6=0,

u=c(0,0,0,0,0,0,0,0,0,0,0,0,0,0,0,0,0,0,0,0,0,0,0,0,0,0,0,0,0,0,0,0,0,0,0,0,0,0,0,0,0,0,0,0,0,0,0),

v=c(0,0,0,0,0,0,0,0,0,0,0,0,0,0,0,0,0,0,0,0,0,0,0,0,0,0,0,0,0,0,0,0,0,0,0,0,0,0,0,0,0,0,0,0,0,0,0))
